# Small is fast: astrocytic glucose and lactate metabolism at cellular resolution

**DOI:** 10.3389/fncel.2013.00027

**Published:** 2013-03-22

**Authors:** L. F. Barros, A. San Martín, T. Sotelo-Hitschfeld, R. Lerchundi, I. Fernández-Moncada, I. Ruminot, R. Gutiérrez, R. Valdebenito, S. Ceballo, K. Alegría, F. Baeza-Lehnert, D. Espinoza

**Affiliations:** ^1^Centro de Estudios CientíficosValdivia, Chile; ^2^Universidad Austral de ChileValdivia, Chile

**Keywords:** FRET, FLII12Pglu-700δμ6, laconic, glycolysis, mitochondria, flux, cancer metabolism

## Abstract

Brain tissue is highly dynamic in terms of electrical activity and energy demand. Relevant energy metabolites have turnover times ranging from milliseconds to seconds and are rapidly exchanged between cells and within cells. Until recently these fast metabolic events were inaccessible, because standard isotopic techniques require use of populations of cells and/or involve integration times of tens of minutes. Thanks to fluorescent probes and recently available genetically-encoded optical nanosensors, this Technology Report shows how it is now possible to monitor the concentration of metabolites in real-time and in single cells. In combination with *ad hoc* inhibitor-stop protocols, these probes have revealed a key role for K^+^ in the acute stimulation of astrocytic glycolysis by synaptic activity. They have also permitted detection of the Warburg effect in single cancer cells. Genetically-encoded nanosensors currently exist for glucose, lactate, NADH and ATP, and it is envisaged that other metabolite nanosensors will soon be available. These optical tools together with improved expression systems and *in vivo* imaging, herald an exciting era of single-cell metabolic analysis.

## Introduction

It is hard to overestimate the importance of resolution. In the absence of sufficient temporal resolution transient events go undetected. Without enough spatial resolution, opposite changes in neighboring compartments may cancel out. Classic biochemistry mapped metabolic pathways and characterized the behavior of purified enzymes in test tubes. Decades before cell sorting, enzymes had to be extracted from whole brain homogenates. Metabolites were measured, again in whole tissue extracts, and a handful of enzymes thought to catalyze far-from-equilibrium reactions were deemed to control flux. With the introduction of radioisotopes in research during the 1950s and non-invasive techniques for their detection, particularly PET and NMRS, concentrations and fluxes could be estimated in living humans, thus permitting biochemical investigation of brain disease. Meanwhile, progress in molecular biology, immunohistochemistry, and the introduction of cell cultures, revealed previously unsuspected complexities, with numerous isoforms for metabolic enzymes and transporters plus cell-specific post-translational modifications. At present, it is evident that neurons and glial cells differ metabolically as much as they differ functionally, but little is known regarding subtypes of neurons and astrocytes and their interaction with oligodendrocytes (Funfschilling et al., 2012; Lee et al., 2012). Of clinical importance are regional variations in metabolism across the brain that may help to explain susceptibility to neurodegeneration (Vaishnavi et al., 2010; Vlassenko et al., 2010; Bero et al., 2011).

Over the last decade, fluorescence microscopy-based techniques with high spatiotemporal resolution have been introduced for the study of energy metabolism in cultured cells and in brain tissue slices. Genetically-encoded sensors are becoming available and it is now possible to measure, glucose, lactate, NADH, and ATP in individual cells with sub-second resolution. The present work describes how some of these sensors may be used in combination with transport inhibitors, to quantify metabolic flux and investigate the regulation of astrocytic glycolysis in response to neuronal activity.

## How local and how fast is brain metabolism?

**Figure 1:**
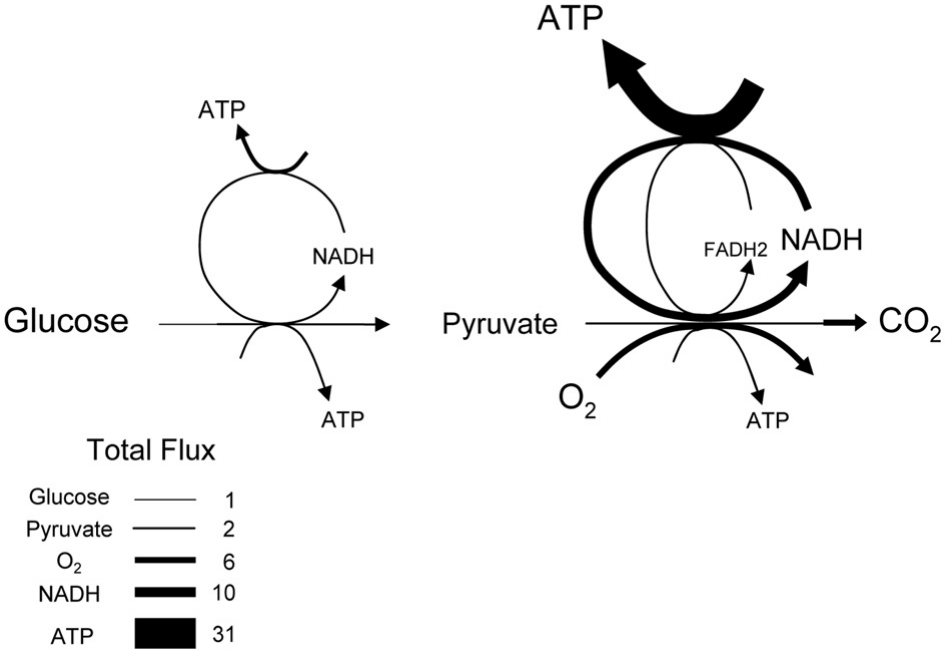
**Stoichiometry of glucose oxidation.** The schematic represents the oxidation of glucose to CO_2_, where the width of the arrows is proportional to flux. Cytosolic NADH is assumed to transfer its electrons to mitochondria through both the malate-aspartate shuttle (rendering 3 ATPs per NADH) and the glycerol phosphate shuttle (rendering 2 ATPs per NADH).

**Figure 2:**
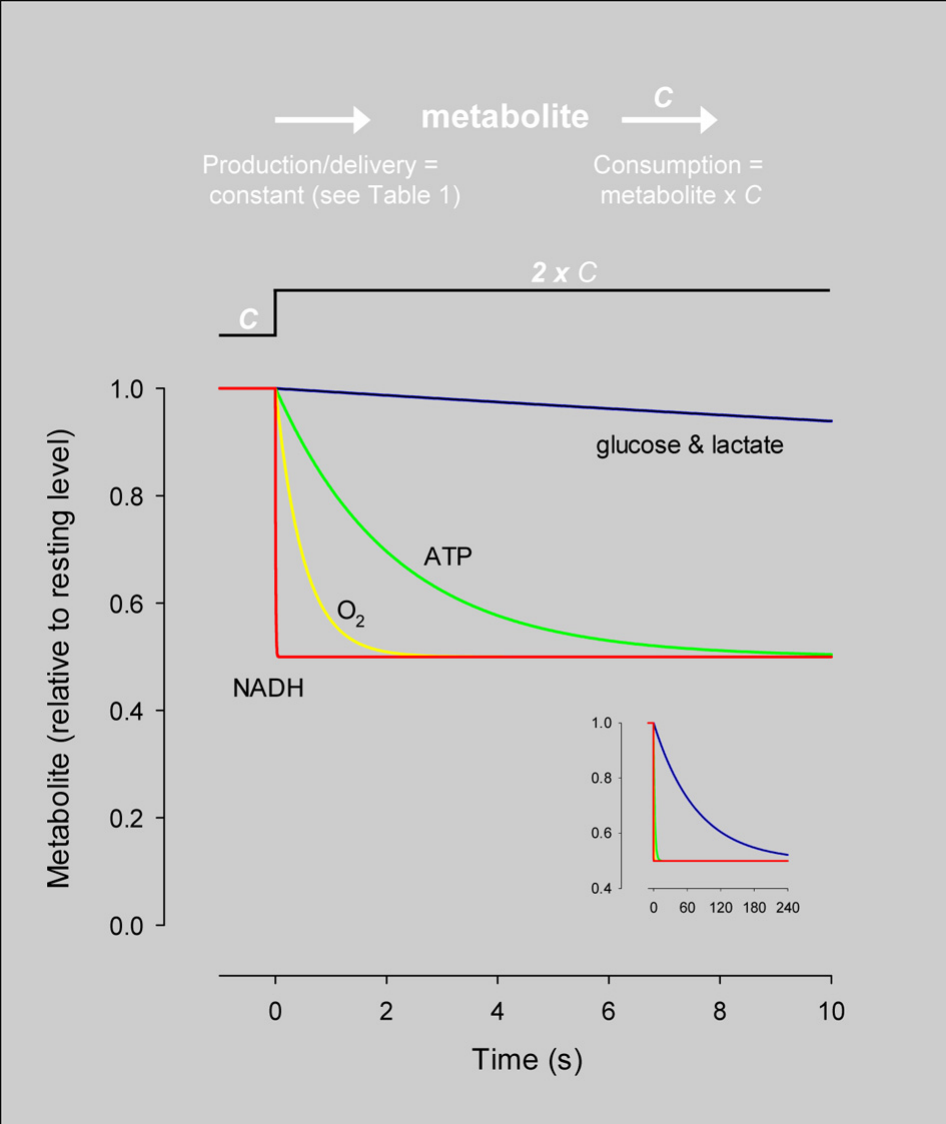
**Simulation of the response of metabolites to an instant rise in consumption.** The dynamics of each metabolite were simulated independently using the concentration and steady-state flux in Table 1 and the differential equation: d metabolite/dt = production–metabolite × *C*, where *C* is the rate constant of consumption. At time zero, *C* was increased by 100% while production was kept constant, resulting in a 50% decrease in the size of the pool. The speed at which the new steady-state is reached varies dramatically between different metabolites. The inset shows the same data over an extended timescale. The differential equation was solved by numerical simulation using Madonna software.

The average rate of glucose utilization in human gray matter has been estimated at 8.8 μM/s (Huang et al., 1980; Gjedde and Diemer, 1983), ten times higher than the body's average. With this value and the known stoichiometry of the glucose oxidation (C_6_H_12_O_6_ + 6O_2_ → 6CO_2_ + 6H_2_O) and coupled reactions, it is possible to obtain an estimate of flux at different points in the metabolic chain. As the glucose molecule proceeds through glycolysis and the Krebs cycle, its free energy is split into smaller packets and the molar flux rises, reaching a maximum at ATP, with 31 molecules produced for each glucose molecule consumed (Figure 1). In addition to flux, metabolite dynamics are determined by concentration, so that the smaller the concentration, the larger the impact of a given flux on the metabolite pool. The ratio between concentration and flux is known as the *turnover time* and is a useful parameter of how dynamic a metabolite is. The turnover time can be thought of as the time that a metabolite pool would last if production were to stop while consumption remained constant. As shown in Table 1, brain tissue glucose and lactate have turnover times in the order of 2 min whereas ATP and oxygen have turnover times of a few seconds, whereas for NADH it is just 7 ms. The turnover time reflect sensitivity of a given metabolite pool to flux perturbation. According to the simulation shown in Figure 2, a 100% increase in the rate of consumption would reduce the respective brain pools with a half time of about 1 min for glucose and lactate, 0.3 and 1.5 s for O_2_ and ATP and 5 ms for NADH. Taken in combination with the diffusion coefficient, the turnover time also helps to reveal how local a metabolite may be if its diffusion were not restricted by membranes. For instance, during its turnover time, the average glucose or lactate molecule can diffuse several hundreds of micrometeres along the cytosol of a neuron, roughly the diameter of a cortical column or a cortical barrel, whereas variations in cytosolic NADH in a dendrite will not be sensed by its soma located just a few micrometers away (Table 1).

**Table 1:** **Dynamics of selected metabolites in brain tissue**.

**Metabolite**	**Glucose**	**Lactate plus pyruvate**	**ATP**	**O_2_**	**NADH**
Stoichiometry^*^	1	2	31	6	2
Concentration (μM)	1000*^a^*	2000*^b^*	1250*^c^*	30*^d^*	0.13*^e^*
Flux*^f^* (μM/s)	8.8	17.6	273	53	17.6
Turnover time*^g^* (s)	114	114	4.6	0.6	0.007
Diffusion coefficient (*D*) (μm^2^/s)	500*^h^*	130*^i^*	500*^j^*	2000*^k^*	500*^l^*
Average distance traveled over 1 and 10 turnover times*^m^* (μm)	585 and 1849	298 and 943	117 and 371	85 and 268	5 and 14
Generation of nanodomains	No	No	No	No	Yes

* *Whole tissue stoichiometry is given for glucose, lactate/pyruvate, ATP and oxygen, while cytosolic stoichiometry is given for NADH*.

a *Human brain tissue (Barros et al., 2007)*.

b *Human brain tissue (Dienel and Cruz, 2004)*.

c *HeLa cells, MIN6 cells, and COSM6 cells (Zamaraeva et al., 2005) and references therein*.

d *Human brain tissue (Buxton, 2010)*.

e *COS7 cells (Zhang et al., 2002)*.

f *The glucose flux was calculated using non-invasive measurements in human gray matter (Huang et al., 1980) and the glucose distribution volume (Gjedde and Diemer, 1983). The flux of the other metabolites was calculated as the product of the glucose flux and the respective stoichiometry. For NADH the cytosolic flux is given*.

g *Turnover time is concentration divided by flux*.

h *Isotopic deoxyglucose in rat vagus nerve (Vega et al., 2003)*.

i *NMRS in rat brain tissue (Pfeuffer et al., 2000)*.

j *NMRS in rat skeletal muscle (de Graaf et al., 2000)*.

k *Oxygen electrode measurements in rat brain tissue (Baumgartl and Lubbers, 1983)*.

l *Assumed to be equal to that of ATP*.

m *Estimated assuming Brownian diffusion according to the Einstein's equation in three dimensions (distance^2^* = 6 × *D* × *turnover time*).

The above considerations help to establish *a priori* the minimum spatial and temporal resolutions required to order to characterize metabolism. Glucose and lactate dynamics have to be sampled in seconds, whereas monitoring ATP and O_2_ may need techniques that resolve hundreds of milliseconds. To avoid missing NADH fluctuations, millisecond sampling will be required. In terms of size, monitoring cytosolic glucose, lactate, O_2_ or ATP demand single-cell resolution, whereas cytosolic NADH and metabolites inside small membrane compartments such as mitochondria demand sub-cellular resolution. One may think that even higher resolution may be needed to characterize the immediate neighborhood of metabolic enzymes and transporters, but this is not the case. Glucose, lactate, O_2_, ATP, and any other molecules present at micromolar levels or higher are not expected to form microdomains or nanodomains, because the build up or depletion of metabolites in the immediate vicinity of the proteins handling these molecules is negligible compared to the powerful mixing effect of diffusion in short distances (Barros and Martinez, 2007; Martinez et al., 2010). For these abundant molecules, the cytosol within an astrocyte or a neuronal soma is expected to behave as a well-mixed compartment. NADH is different, because its cytosolic concentration is very low. For example, considering a cytosolic NADH of 130 nM (*u*), a diffusion coefficient (*D*) of 500 μm^2^ s^−1^ (Table 1), and that a single lactate dehydrogenase enzyme (LDH) produces or consumes lactate at a rate of 260 s^−1^ (*q*, Barros and Martinez, 2007), the relative amplitude (*AMP*) of the local NADH nanodomain may be estimated using the equation *AMP = 1 ± q*/(*u × D × a*), where *a* is the radius of the catalytic site (Martinez et al., 2010). According to this formula and assuming a radius of 0.5 nm (Barros and Martinez, 2007), LDH is predicted to create a local nanodomain in which the concentration of NADH is twice that of the bulk cytosolic NADH when the enzyme is consuming lactate, whereas LDH should deplete its vicinity of NADH when the enzyme is producing lactate. Thus, an accurate characterization of NADH dynamics will require nanometer resolution.

## Fast glucose dynamics measured with fluorescent glucose analogs

2-deoxyglucose is a glucose analog that is transported into cells by the GLUT glucose transporters and then phosphorylated by hexokinase, but is not metabolized further to any significant extent. Detected in cultured cells by scintillation counting, by autoradiography in laboratory animals and non-invasively in humans with FDG-PET, radiolabeled 2-deoxyglucose has wide applications in research and clinical medicine. However, like other radioisotopes, it has limited spatiotemporal resolution, with detection requiring cell populations and typical sampling intervals in excess of 10 min.

Fluorescent analogs of glucose have been used to characterize the transport and metabolism of glucose at high resolution by means of microscopy (Kim et al., 2012). The most popular analogs are 2-NBDG and 6-NBDG. These compounds are comprised of a glucose moiety in which a fluorescent nitrobenzoxydiazoamine (NBD) group replaces the hydroxyl group at carbon 2 or 6. Both are substrates of GLUT carriers but only 2-NBDG can be phosphorylated by hexokinase. As demonstrated with 6-NBDG, the bulky hydrophobic NBD group increases the affinity of binding to GLUTs, but impairs translocation of the binding site to a larger extent (Barros et al., 2009a), making transport of 6-NBDG by GLUT1 and GLUT3, respectively 100 and 16 times slower than that of glucose (Jakoby et al., 2012). The low efficiency of translocation provides an important experimental advantage because it permits the use of confocal microscopy to monitor uptake in real time over a period of several minutes, a time window in which agonists can be applied to investigate acute modulation of glucose transporters (Loaiza et al., 2003; Porras et al., 2004, 2008). These fluorescent glucose tracers have also been used to characterize glucose uptake in many other mammalian cell types including erythrocytes, fibroblasts, smooth muscle cells, enterocytes, cardiomyocytes, endothelium, lymphocytes, pancreatic beta cells, adipocytes, and tumor cells (Barros et al., 2009a; Kim et al., 2012). Imaging of 2- and 6-NBDG by multiphoton microscopy has been used to study the transport and metabolism of glucose in cerebellar and hippocampal slices (Barros et al., 2009b; Jakoby et al., 2012) and to detect a stimulatory effect of neural activity on glucose transport in astrocytes in the somatosensorial cortex *in vivo* (Chuquet et al., 2010). Long-term (>10 min) incubation with 2-NBDG followed by a washout period to remove unphosphorylated 2-NBDG is informative about glucose consumption, but 2-NBDG cannot be used to monitor metabolism in real time, because both the phosphorylated and unphosphorylated form of the analog are fluorescent, making it impossible to differentiate between the two. However, single-cell real-time monitoring of glucose metabolism is now possible with a genetically-encoded FRET glucose nanosensor.

## Glucose metabolism measured with a genetically-encoded FRET nanosensor

**Figure 3:**
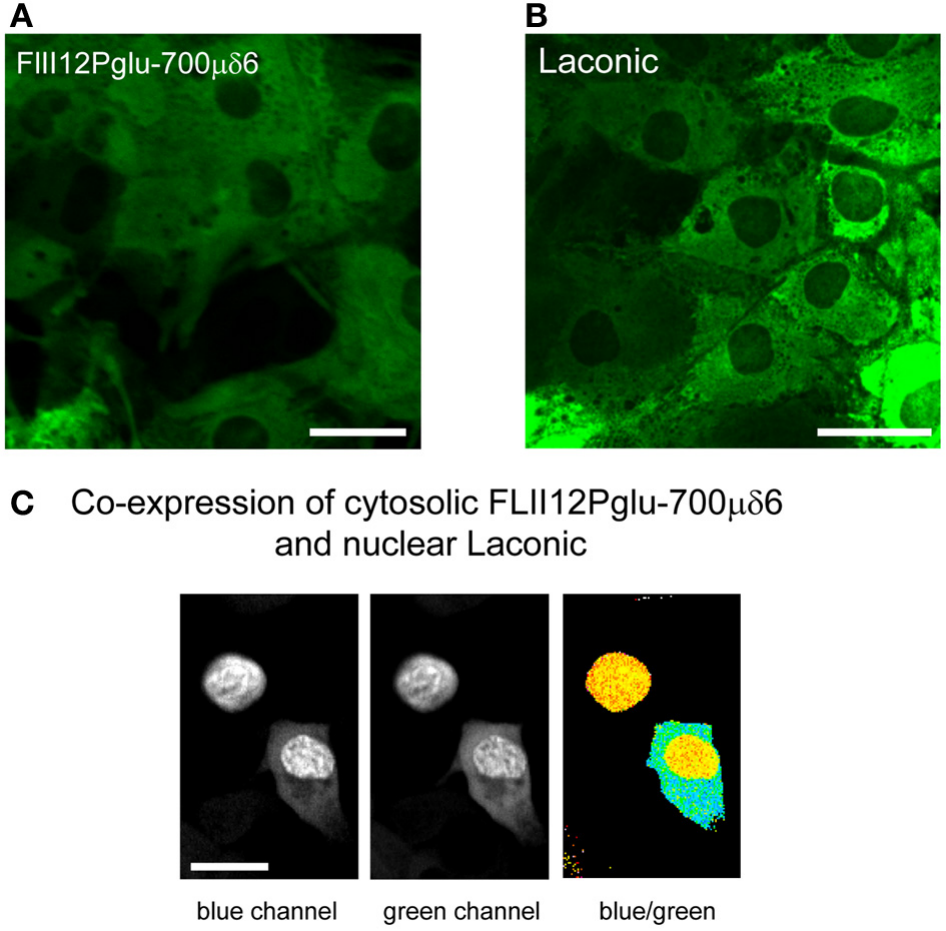
**Expression of the glucose and lactate sensors in astrocytes and HEK293 cells. (A,B)** The FRET glucose nanosensor FLII12Pglu700μδ6 and the FRET lactate nanosensor Laconic were expressed in astrocytes using a custom-made adenoviral vector (Vector Biolabs). Confocal images correspond to green emission at 535 nm (Venus) as excited with a 488 nm argon laser. Bar represents 20 μm. **(C)** HEK293 cells were co-transfected with FLII12Pglu700μδ6 and nuclear-targeted Laconic. The confocal images show the CFP and mTFP emissions at 480 nm (blue channel) and the Citrine and Venus emissions at 535 nm (green channel) of a cell expressing only Laconic (top) and a cell expressing both sensors (bottom). Scale bar is 20 μm.

Ten years ago Wolf Frommer and colleagues introduced the first FRET glucose nanosensor (Fehr et al., 2003), making an improved version available in 2008 (Takanaga et al., 2008). Since then, various research groups have made fluorescent nanosensors specific for ATP (Berg et al., 2009; Imamura et al., 2009) and NADH (Hung et al., 2011; Zhao et al., 2011) and we have developed a FRET nanosensor for lactate (San Martín et al., 2013). The glucose and lactate nanosensors are of the same principle. They comprise a bacterial protein that binds the analyte, sandwiched between two fluorescent proteins with overlapping emission and excitation spectra that undergo FRET. Binding of the analyte to the bacterial protein induces a conformational change that modifies the distance between the fluorescent protein and/or its relative orientation, resulting in a change in FRET efficiency which can be calibrated. Figures 3A,B shows cultured astrocytes expressing the glucose nanosensor (FLII12Pglu-700μδ6) and the lactate nanosensor (Laconic), with the typical cytosolic distribution and exclusion of nuclei and organelles. In Figure 3C, Laconic has been targeted to the nucleus and FLII12Pglu-700μδ6 to the cytosol of HEK293 cells, which permits the use of confocal microscopy to simultaneously monitor glucose and lactate in the same cell.

**Figure 4:**
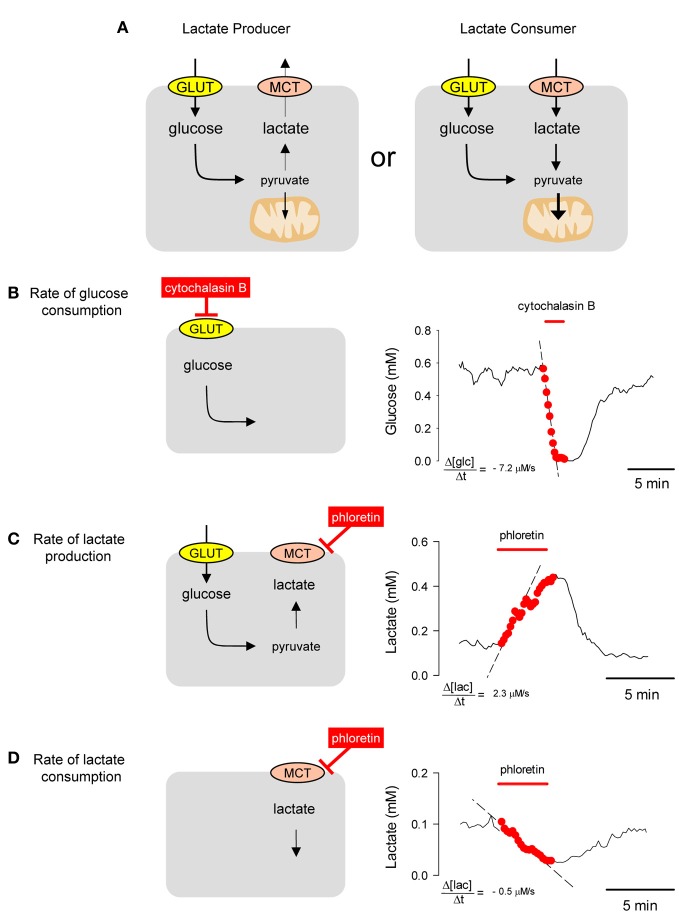
**Estimation of metabolic fluxes with transport-stop protocols.(A)** Whereas most mammalian cells are glucose importers, some cells export lactate and some import lactate. **(B)** A single astrocyte incubated in 2 mM extracellular glucose maintained an intracellular glucose of approx. 0.6 mM. Interruption of the steady state by blocking the glucose transporter GLUT1 with 20 μM cytochalasin B caused a decrease in the cytosolic concentration of glucose at a rate of −7.2 μM/s. **(C)** A HEK293 was incubated in 25 mM glucose. Blockage of the MCT with 50 μM phloretin caused an accumulation of intracellular lactate with an initial rate of 2.3 μM/s. **(D)** Same cell as in **(B)** but incubated in 1 mM lactate in the absence of glucose. Blockage of the MCT with 50 μ M phloretin caused a depletion of intracellular lactate with an initial rate of −0.5 μM/s.

All mammalian cells metabolize glucose but they differ in their handling of lactate. Some cells are lactate exporters, while others are lactate importers (Figure 4A). The concentration of a metabolite, absolute or relative, may be interesting in itself, as it informs about the balance between production and consumption (Fehr et al., 2003; Bittner et al., 2010, 2011; Takanaga and Frommer, 2010; Kovacic et al., 2011; Prebil et al., 2011). However, concentration gives no information about flux. For instance, a decrease in intracellular glucose may be due to an inhibition of GLUT-mediated transport (with flux decrease) or a stimulation of hexokinase (with flux increase). Moreover, the rate of departure from the steady-state is sensitive to the degree of GLUT or hexokinase modulation but equally sensitive to resting flux (Barros et al., 2013), with a fast cell reacting more quickly than a slow cell to the same degree of stimulation (Barros et al., 2013). However, by eliminating the contribution of transport with a GLUT blocker like cytochalasin B, the ambiguity is lifted as the glucose concentration is forced to decrease with a rate equal to that of glucose consumption (Figure 4B). This is a protocol that can be applied repeatedly and which has been validated in astrocytes, neurons, muscle cells, fibroblasts, adipocytes, and tumor cells (Bittner et al., 2010). Most cells express a high affinity isoform of hexokinase, with a K_D_ of 50 μM, and in the presence of millimolar extracellular glucose maintain steady-state intracellular glucose levels of 0.5 mM or higher, so that hexokinase runs at V_max_. This explains the linear decrease in glucose concentration during the GLUT block. When applied to astrocytes in culture and in organotypical hippocampal slices, this GLUT-stop technique revealed that glycolysis is strongly and reversibly stimulated within seconds of exposure to elevated extracellular K^+^, a cation that is released by active neurons (Bittner et al., 2011). Further work showed that K^+^ activates astrocytic glycolysis by a sequence of events that begin with plasma membrane depolarization, then stimulation of the electrogenic Na^+^/bicarbonate co-transporter NBCe1 and intracellular alkalinization (Ruminot et al., 2011). We also confirmed the stimulatory effect of glutamate on glycolysis that was detected two decades ago by Pellerin and Magistretti using 2-deoxyglucose (Pellerin and Magistretti, 1994; Pellerin et al., 2007) and showed that the effect of glutamate on glucose consumption develops over minutes and persists long after withdrawal of the neurotransmitter (Bittner et al., 2011). More recently, the same method detected fast changes in the rate of astrocytic glycolysis in response to variations in extracellular lactate (Sotelo-Hitschfeld et al., 2012), a phenomenon that may be relevant for the local distribution of fuel in brain tissue. A general protocol for the use of FRET nanosensors for metabolites can be found in Hou et al. (2011) and a more specific practical guide to the use of the glucose sensor for quantification of glucose consumption is given by Barros et al. (2013).

## Lactate dynamics measured with a genetically-encoded FRET nanosensor

*LACtate Optical Nano Indicator from Cecs* (Laconic) is comprised of the *Escherichia coli* protein LldR, the teal fluorescent protein mTFP and the green fluorescent protein Venus (San Martín et al., 2013). In bacteria, LldR regulates the transcription of an operon required for the metabolism of lactate. The binding of lactate to Laconic is best described by a two-site model, having a high-affinity component with a K_D_ of 8 μM and a low-affinity component with a K_D_ of 800 μM, with each accounting for about 50% of the change in the fluorescence ratio. Expressed in mammalian cells, the maximum change in fluorescence ratio is about 40%. The sensor is insensitive to the NADH/NAD^+^ ratio and physiological levels of pyruvate and other acidic metabolites. It shows little sensitivity to pH changes in the physiological range of the mammalian cytosol, (pH 7.0–7.4) (San Martín et al., 2013).

In mammalian cells, lactate is transported across the plasma membrane by the monocarboxylate transporter (MCT). Use of the lactate sensor to estimate the flux of lactate in HEK293 cells with an MCT-stop protocol is illustrated in Figures 4C,D. In the presence of glucose, exposure of cells to the MCT inhibitor phloretin caused an increase in the concentration of intracellular lactate, showing that the cell was exporting lactate. The rate of lactate accumulation reached a maximum immediately after MCT blockage and reflects the magnitude of the lactate flux in the steady-state, decreasing later because feedback inhibition of glycolysis by lactate (Sotelo-Hitschfeld et al., 2012) and/or delayed depletion of intracellular glucose caused by phloretin, also a GLUT blocker. Similar results have been obtained with the non-permeant MCT blocker pCMBS and with the more specific MCT blocker AR-C155858 (San Martín et al., 2013). Inhibition of the MCT in the same HEK293 cells incubated in the presence of lactate but with glucose absent, caused a decrease in intracellular lactate, showing that the cell was importing lactate. This type of protocol may be used to address the question of intercellular lactate exchange, which is of great interest for neuroenergetics (Pellerin et al., 2007; Barros and Deitmer, 2010; Allaman et al., 2011; Wyss et al., 2011) and in cancer research (Yeluri et al., 2009; Tennant et al., 2010).

**Figure 5:**
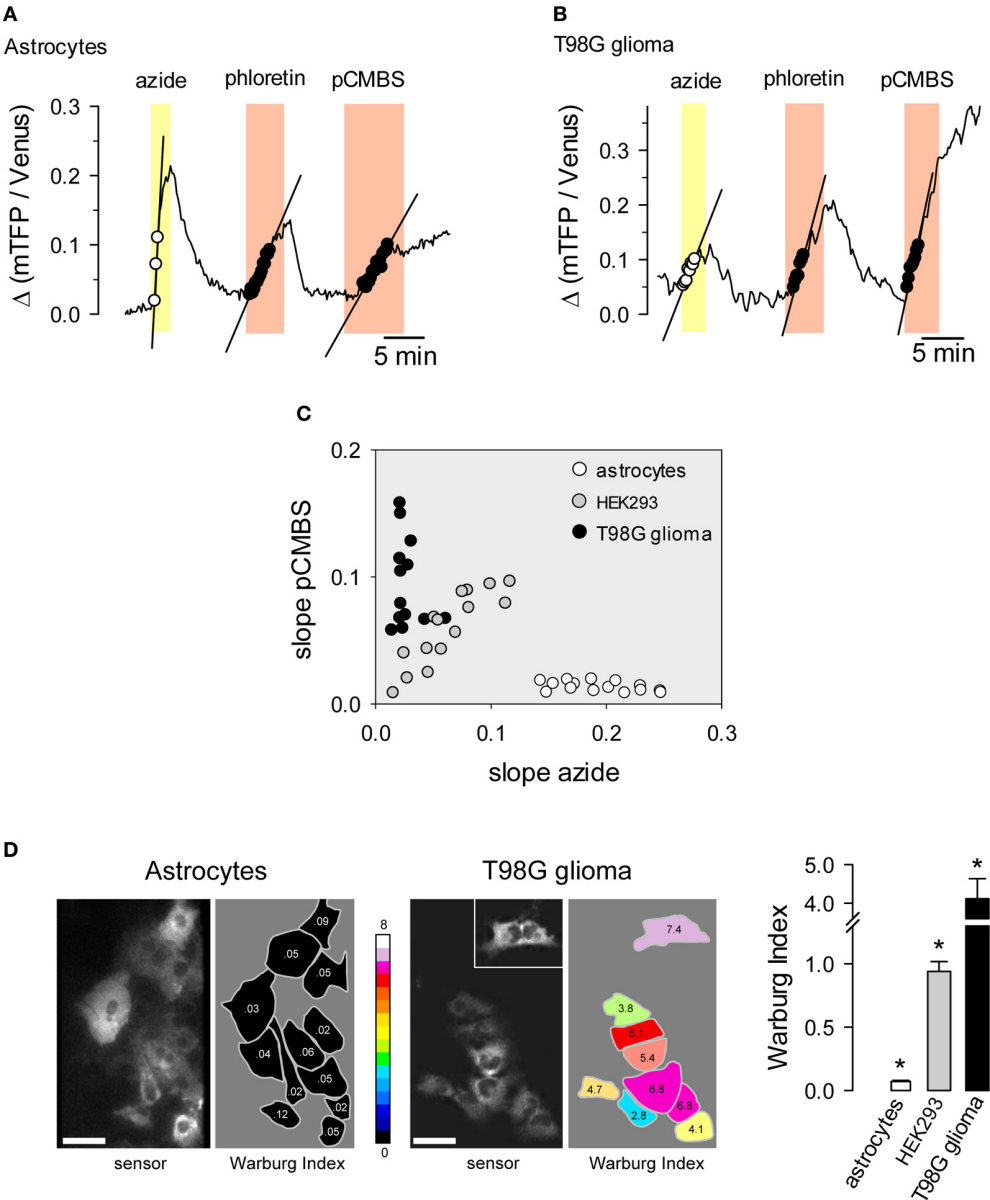
**Use of the lactate sensor to detect the Warburg effect.** An astrocyte **(A)** and a T98G glioma cell **(B)** expressing Laconic were sequentially exposed to 5 mM sodium azide, 50 μM phloretin and 500 μM pCMBS. The straight lines represent initial slopes of lactate accumulation fitted by linear regression within the same range of ratio values. **(C)** Correlation plot between the rates of lactate accumulation (Δ ratio/min) in sodium azide and in pCMBS. Symbols represent single astrocytes (white), HEK293 cells (gray) or T98G cells (black). **(D)** The Warburg Index was estimated as the ratio between the rates of lactate production with pCMBS and lactate accumulation with azide, and was used to color the silhouette of each cell according to the 16-color look up table. The inset shows an isolated cell that was located about 100 μm from the cluster. The bar graph summarizes data from 3 experiments in each cell type. Scale bars are 20 μm. ^*^p < 0.05 between every cell type. Modified from San Martín et al. (2013).

Normal cells oxidize most of the glucose they take up, exporting small amounts of lactate or importing lactate. Cancer cells often have deficient mitochondria and a strong glycolytic flux, exporting much more lactate than normal cells. This phenomenon is known as the Warburg effect and may be important for cancer progression (Vander Heiden et al., 2009). The Warburg effect can be detected non-invasively in humans by FGD-PET for the purposes of cancer diagnosis and staging. For basic research, the Warburg effect may be observed in cell populations or tissue explants by monitoring the rate of oxygen consumption. Taking advantage of the resolution afforded by the FRET lactate sensor, we devised a protocol that provides a quantitative estimate of the Warburg effect in single cells (San Martín et al., 2013). Cells are first exposed to sodium azide, a reversible inhibitor of oxidative phosphorylation. Normal cells respond to mitochondrial poisoning with an acute stimulation of glycolysis (Bittner et al., 2010), causing a rapid accumulation of intracellular lactate (Figure 5A). This response is weaker in cancer cells (Figure 5B). After intracellular lactate returns to baseline levels, cells are exposed to an MCT blocker in order to measure the rate of lactate production, which is higher in cancer cells. The difference between astrocytes and T98G glioma cells becomes evident when both rates are plotted for each cell as shown in Figure 5C. The difference can be quantified by computing the ratio between the rate of lactate production and the rate of lactate accumulation with sodium azide, termed the Warburg Index (WI), as illustrated in Figure 5D. Astrocytes had WI values of <0.1 whereas T98G glioma cells had WI values of >2, with intermediate values observed for the non-transformed cell line HEK293. Tumors are complex systems in which cancer cells of differing degrees of malignancy co-exist with non-cancerous cells of several lineages. The single-cell resolution provided by genetically-encoded FRET nanosensors together with multiphoton microscopy may help to investigate metabolic exchanges within tumors.

## Conclusion and perspectives

Genetically-encoded sensors permit single cell estimation of metabolite concentration with sufficient temporal resolution to detect physiological fluctuations. In the case of glucose and lactate, inhibitor-stop protocols are available that measure rates of usage and production. Together with improved expression systems and imaging in tissue slices and *in vivo*, these tools open the way to a characterization of energy metabolism in identified cells in healthy and diseased tissue.

### Conflict of interest statement

The authors declare that the research was conducted in the absence of any commercial or financial relationships that could be construed as a potential conflict of interest.
